# Formation and Propagation of Tau Oligomeric Seeds

**DOI:** 10.3389/fneur.2013.00093

**Published:** 2013-07-17

**Authors:** Julia E. Gerson, Rakez Kayed

**Affiliations:** ^1^George P. and Cynthia Woods Mitchell Center for Neurodegenerative Diseases, University of Texas Medical Branch, Galveston, TX, USA; ^2^Department of Neurology, University of Texas Medical Branch, Galveston, TX, USA; ^3^Department of Neuroscience and Cell Biology, University of Texas Medical Branch, Galveston, TX, USA

**Keywords:** tauopathies, tau oligomers, oligomeric seeding, propagation of tau pathology, Alzheimer’s

## Abstract

Tau misfolding and aggregation leads to the formation of neurofibrillary tangles (NFTs), which have long been considered one of the main pathological hallmarks for numerous neurodegenerative diseases known as tauopathies, including Alzheimer’s Disease (AD) and Parkinson’s Disease (PD). However, recent studies completed both *in vitro* and *in vivo* suggest that intermediate forms of tau, known as tau oligomers, between the monomeric form and NFTs are the true toxic species in disease and the best targets for anti-tau therapies. However, the exact mechanism by which the spread of pathology occurs is unknown. Evidence suggests that tau oligomers may act as templates for the misfolding of native tau, thereby seeding the spread of the toxic forms of the protein. Recently, researchers have reported the ability of tau oligomers to enter and exit cells, propagating from disease-affected regions to unaffected areas. While the mechanism by which the spreading of misfolded tau occurs has yet to be elucidated, there are a few different models which have been proposed, including cell membrane stress and pore-formation, endocytosis and exocytosis, and non-traditional secretion of protein not enclosed by a membrane. Coming to an understanding of how toxic tau species seed and spread through the brain will be crucial to finding effective treatments for neurodegenerative tauopathies.

## Tau Oligomers are the Toxic Tau Species in Neurodegenerative Tauopathies

The formation of tau aggregates and neurofibrillary tangles (NFTs) is one of the main pathological hallmarks of numerous diseases termed tauopathies, including the two most common neurodegenerative diseases, Alzheimer’s Disease (AD) and Parkinson’s Disease (PD) (1, 2). However, it is evident that cell death occurs initially prior to NFT formation in AD (3–6) suggesting that NFTs are not the pathogenic species responsible for the spread of the disease. Recent evidence points to the presence of multimeric tau species which are intermediates between tau monomers and NFT – known as tau oligomers – as the toxic species inducing synaptic dysfunction and cell death in neurodegenerative tauopathies (7–12).

Numerous researchers have investigated tau pathology using animal models, yielding a better understanding of the toxicity of different tau structures. A study in aged mice expressing native human tau (htau mice) found that while NFT formation occurred as animals aged, there was no correlation between the presence of tau filaments and cell death (13). Additionally, a study examining the P301S mouse model, which expresses mutant human tau, found that hippocampal synaptic dysfunction occurred prior to NFT formation (14). Studies using the rTg4510 mouse model, which conditionally expresses P301L mutant tau, found that cell death occurred prior to NFT formation and that cell loss and behavioral impairments could be suppressed by inhibiting tau expression without removing NFTs or preventing their continued accumulation (7, 15). In accordance with this finding, it has been shown that NFTs are protective in the same mouse model (16), and only pro-aggregate human tau mice (TauRD) show behavioral deficits (17). Another study in the same mouse model characterized tau oligomers biochemically that appeared early and correlated with cognitive deficits (8, 12). Similar results have also been seen in *Drosophila* AD models, where expression of mutant tau causes neurodegeneration, synaptic dysfunction, and axonal transport deficiencies in the absence of NFTs (18, 19). Usage of the protein nicotinamide mononucleotide (NAD) adenylyl transferase (NMNAT) was shown to decrease behavioral and morphological deficiencies in a frontotemporal dementia *Drosophila* model by decreasing levels of tau oligomers (20).

**Figure 1:**
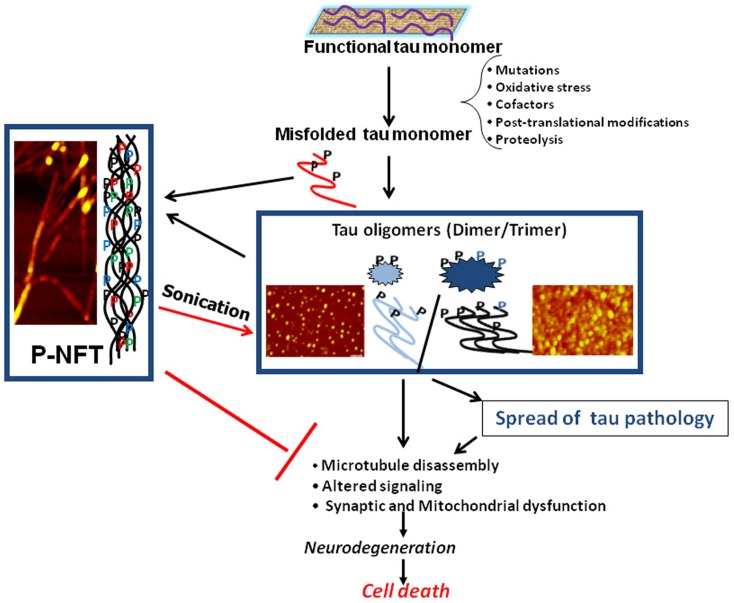
**Schematic illustrating the central role of tau oligomers in tauopathies**. Tau intermediate soluble aggregates (tau oligomers) are the toxic tau entities and initiators of tau pathology and propagation in tauopathies, rather than monomeric tau or hyperphosphorylated NFTs (p-NFTs). Sonication of fibrillar tau generates toxic tau oligomers. Thus, tau oligomers represent the ideal target for anti-tau therapeutic approaches. AFM images are of brain-derived tau oligomers and NFT (72, 138).

Biochemical analysis of human AD brain tissue has also yielded results suggesting that tau oligomers may initiate toxicity, rather than NFTs. When compared to control brains, levels of tau oligomers were found to be significantly increased in AD brains early in the disease, prior to when NFTs appear and clinical symptoms are evident (9, 21–23). In addition to correlative evidence for the importance of tau oligomers to toxicity, treatment with tau oligomers has also been shown to cause adverse effects in animals. Isolated tau oligomers, but not monomers or NFTs, induced memory impairments, synaptic dysfunction, and mitochondrial dysfunction when given intracerebrally to wild-type mice (24). Therefore, it is possible that NFTs are actually neuroprotective, sequestering toxic forms of tau into large aggregates with less flexibility and surface area to interact with cells. All of these studies form the framework for the model of the progression of neurodegenerative tauopathies beginning with the seeding and propagation of toxic tau oligomers (Figure 1).

## Tau Oligomers are Seeds for the Propagation of Pathological Tau

Recently, researchers have begun to make comparisons between the spread of neurodegenerative disease and prion disease, as studies suggest that misfolded protein templating, known as seeding, may underlie the progression of disease (25). Understanding how tau seeds pathological forms of the protein which propagate to different brain regions is critical to devising a solution to stop the spread of disease. There are two main models for the formation and seeding of pathological tau, oligomer-nucleated conformational induction – based upon the mechanism of action of prion protein, Sup35 (26) and amyloid β (Aβ) (27) – and template-assisted growth. Template-assisted growth proposes that tau fibrils act as template molecules for unfolded monomers. When monomers come in contact with filaments, they are integrated into the filament in organized, parallel stacked β sheets, optimizing hydrogen bonding for stabilization (28). It has been difficult, however, to find spontaneous tau aggregation which occurs experimentally. When fibrils are cleaved, leaving only three microtubule binding repeats, the fragments aggregate spontaneously *in vitro* (29). However, on its own, tau will not polymerize *in vitro* without the addition of certain reagents, post-translational modifications, such as phosphorylation, or induction of mutations.

In order for aggregation to occur, tau must be released from microtubules to reach a high concentration of free cytosolic tau, conformational changes must occur to allow for aggregation, possibly by increasing β sheet content, and dimerization must occur (30, 31). The addition of polyanions, such as heparin or RNA can induce fibrillization of tau (32), causing a conformational change from random coil structure to β sheet structure (33). Free fatty acids, such as arachidonic acid can also increase aggregation (34, 35) due to the presence of an alkyl chain, which induces micellization, and a negatively charged head group on the fatty acid to create a negatively charged surface on the micelle. In the presence of tau, the critical concentration for micelle formation is greatly decreased, allowing anionic micelles to attract tau to the negatively charged surface and thereby compensate for positive charges in tau and enable tau aggregation (36, 37). Phosphorylation may also play a role in fibrillization. Paired helical filaments (PHF) and straight filaments (SF), which make up the NFTs found in the brains of patients with AD, are comprised of hyperphosphorylated tau (38, 39). Phosphorylated tau has a higher tendency toward aggregation than unphosphorylated tau and kinases involved in the phosphorylation of these sites in tau have been shown to be altered in AD (40). Hyperphosphorylated tau has been shown to aggregate *in vitro*, possibly due to the addition of negative charge which would increase aggregation, similarly to the addition of polyanions and free fatty acids. Furthermore, this process can be inhibited by dephosphorylation (41, 42). Phosphorylation may also induce aggregation by reducing the interaction of tau with microtubules and allowing it to interact instead with unphosphorylated tau and form aggregates (43–45). Mutations, such as those that lead to frontotemporal lobar degeneration (FTLD), can increase tau aggregation through different mechanisms. Many mutations lead to a decrease in microtubule assembly kinetics, which could lead to more free cytosolic tau and increase aggregation (46–49). Some mutations lead to a decrease in the dissociation constants (*K*_d_) for dimer and filament formation, while others increase the rate of nucleation without affecting *K*_d_ (30). Mutations that cause increased formation of β-sheets lead to heightened aggregation due to an increase in hydrophobic interactions, deviating from the highly hydrophilic native tau protein (50, 51). Fibrillar tau can thereby be recognized by dyes which interact with β-sheets, such as Congo Red and thioflavin S (52). However, at high concentrations, these dyes can induce fibrillization due to an attraction between positive charges formed in the core of PHFs and negative charges of the anionic dyes (53–55).

In amyloid proteins in which seeding has been well-established, such as prion proteins and Aβ, oligomers have been shown to be the most potent seeds (56, 57), working by way of oligomer-nucleated conformational induction (26, 27). Due to the increased interest in the toxicity of tau oligomers, evidence has emerged in support of the oligomer-nucleated conformational induction model as more studies have begun to explore the importance of tau oligomers in the initialization of tauopathies. Oligomer-nucleated conformational induction entails oligomers or conformational changes irreversibly stabilizing the highest energy protein states, known as the nucleus, allowing stable monomers to aggregate into oligomeric structures. Oligomers are driven to further elongation to form lower energy, stable filaments (58). As opposed to template-assisted growth, monomers are not incorporated directly into fibrils, but are instead entirely aggregated into oligomers prior to filament formation (59). Tau dimerization increases the tendency to aggregate and can be induced by oxidation (60), which suggests that tau oligomerization may be an important step in the fibrillization pathway. The appearance of oligomeric species of other amyloid proteins has been observed on the path to fibril formation (61–63).

While the addition of reagents and mutations used to induce fibrillization has been integral to understanding how tau aggregation occurs, it does not explain how fibril formation may occur spontaneously in sporadic disease. The mechanism by which tau aggregation occurs physiologically has not yet been elucidated, however there have been some advances in the understanding of how certain steps in the process may occur. Release of tau from microtubules may occur following post-translational modifications, such as phosphorylation (43, 44). Localization to anionic surfaces, alternative splicing, and post-translational modifications stabilizing aggregated conformations may all act as enhancers to increase speed of nucleation (64). Under physiological conditions, nucleating cofactors can induce tau aggregation in a similar fashion to agents used *in vitro*. There is evidence that polyanionic species, such as tubulin, RNA, and α-synuclein can increase the tendency of tau to aggregate (65–67) The formation of disulfide bridges is critical for the initial creation of dimers from monomers, as well as intermolecular crosslinking of the microtubule binding domain independent of cysteine to continue oligomerization of three-repeat tau (68). Prior to monomer aggregation into oligomers, the free energy of solvation decreases, causing a shift in preference for peptide-solvent interactions toward peptide–peptide interactions, as water is evacuated due to poor interaction with the peptide backbone and sidechains. Water release increases entropy of the solvent, thereby balancing the loss in conformational entropy caused by aggregation. The interaction of side chains with the backbone in the form of hydrogen bonding leads to the creation of β-sheet structure and aggregate stabilization. While oligomers form a similar structure to fibrils, they are not as ordered, which likely increases their toxicity (69). Proteolytic processing by endogenous proteases has also been shown to create self-aggregating fragments, which nucleate and co-aggregate with full-length protein effectively enough for a small amount of fragment to seed PHFs (70). Direct interactions between misfolded tau and native protein may be the underlying mechanism of seeding as experiments have shown tau protein–protein interactions occur when tau aggregates enter cells containing native tau (71).

Tau oligomers – identified with the tau oligomer-specific antibody, T22, which does not recognize monomers or fibrils (59) – which have been seeded with oligomers derived from brain tissue have been shown to be highly toxic (23, 72). When tested with Bis-ANS, which recognizes exposed hydrophobic patches, oligomers had higher affinity for Bis-ANS than PHFs, which may underlie toxicity. The toxic effects of tau oligomers formed by seeding recombinant tau with oligomeric seeds, however, can be prevented when pre-treated with T22 (23, 72).

Some tauopathies, such as progressive supranuclear palsy (PSP), only have one pathogenic species involved in disease progression (1). However, most tauopathies contain other amyloid proteins in addition to tau, such as Aβ in AD and α-synuclein in PD. In such diseases, cross-seeding of heterologous protein species is an additional mechanism which is important for tau seeding (59, 67, 73–77).

## Tau Oligomers Propagate from Affected Brain Regions to Unaffected Regions

Aβ has been shown to propagate from affected brain areas to unaffected areas in mice over-expressing Aβ precursor protein that have been injected with Aβ isolated from the brains of AD patients and AD transgenic mice (78, 79), suggesting that perhaps tau could spread in a similar fashion. A few years later, a similar mechanism was demonstrated for the propagation of tau. When tau extracted from P301S mice was injected into the brains of mice over-expressing wild-type human tau (ALZ17 mice), which do not form tau aggregates, tau pathology was observed to have spread from the injection site to neighboring brain regions (80). Additionally, in transgenic mice which differentially express pathological tau in the entorhinal cortex, where tau pathology is first observed in AD, human tau has been shown to spread to both neighboring and synaptically connected neurons which do not express human tau mRNA. Translocated human tau was able to seed mouse tau misfolding (81, 82). However, these studies did not specifically investigate which tau species specifically induced seeding and propagation of tau pathology and the usage of transgenic mouse models is not analogous to sporadic forms of AD. When wild-type mice were injected with both tau oligomers and PHFs isolated from AD brains, tau oligomers induced the spread of tau pathology from the injection site to neighboring brain regions and impaired memory, as measured by object recognition. Conversely, mice injected with PHFs only exhibited tau pathology near the injection site and did not exhibit any memory impairments on the behavioral task, suggesting that tau oligomers, but not fibrillar tau, is capable of seeding and propagating pathology (72). Furthermore, similar results have been found using primary neurons. Neurons were exposed to low molecular weight aggregates – recognized by the tau oligomer-specific antibody, T22, and examined via electron microscopy for oligomeric characteristics – as well as to fibrils formed *in vitro*, filaments formed *in vivo*, and monomers. Low molecular weight aggregates and short fibrils exhibited uptake into the cell, but monomers and filaments were not internalized (83). Other studies have shown tau aggregate uptake using cell culture, but did not specifically identify the type of aggregates being internalized. Neural stem cells treated with tau monomers and aggregates formed using the tau microtubule binding repeat region induced to fibrillize with arachidonic acid, exhibited significantly more tau aggregate uptake than monomer uptake. Additionally, aggregates, but not monomers, induced seeding of endogenous tau misfolding (84).

On the other hand, Guo and Lee hypothesized that seeding of pathological tau in cultured cells would be able to occur more quickly by seeding with pre-formed tau fibrils, thereby omitting the step where monomer must be converted to oligomer prior to fibril formation. Fibrillization of recombinant tau was induced with the addition of heparin and was verified using thioflavin T. Fibril-treated cells exhibited seeding and propagation of aggregates via endocytosis. However, following fibril confirmation with thioflavin T and prior to cell treatment, fibrils used in this study were sonicated (85). Previous research investigating Aβ seeding found that sonication increased seeding ability by fragmenting fibrils into smaller, soluble species (57) and sonicated prions have also been shown to have more potent seeding potentials than unsonicated fibrils (56). Since it has been shown that both prion and Aβ oligomers, rather than fibrils, are the seeds for pathological protein templating (26, 27), it is likely that sonication partially converts insoluble fibrils into soluble oligomeric forms. Sonication of tau fibrils has also been shown to cause shearing of filaments, particularly those in PHF form (86). Therefore, it is likely that sonicated tau fibrils used to treat cells in the previous study (85) also contained tau in oligomeric form, which may explain why seeding and propagation was successful.

Recently, Wu et al. studied propagation of tau in primary neurons using microfluidic chambers which allow somatodendritic compartments to be isolated from axonal compartments, enabling not only the analysis of tau uptake from the extracellular space into the cell, but also propagation within the neuron. They found that low molecular weight tau aggregates specifically recognized by tau oligomer-specific antibody, T22, propagate between isolated neuronal compartments both anterogradely and retrogradely (83). Importantly, tau is primarily found in the axons of healthy neurons (87), though tau may also be found in the dendrite where it colocalizes with the src kinase, fyn (88, 89). In AD, however, misfolded and hyperphosphorylated tau accumulates in the axon, dendrites, and the cell body (90), suggesting that intracellular transport may also be important for the spread of disease. In lamprey neurons expressing low levels of tau, tau was primarily localized to the axon and proximal dendrites, both regions consistent with tau functioning as a microtubule-associated protein. However, in neurons expressing high levels of tau, tau was found in distal dendrites and near the soma membrane, both areas lacking microtubules. High-expressing tau cells showed more degeneration and secretion of tau. Moreover, as tau can modulate activity of microtubule-associated motor proteins involved in dendritic transport, tau localized to the dendrite may have implications for its propagation (91). Phosphorylated tau localized at the synapse in AD brain samples appears to correlate with ubiquitin-proteasome system (UPS) dysfunction, suggesting that tau oligomer accumulation at the synapse impairs the UPS, which is a crucial player in the breakdown of tau. Accumulation of tau at the synapse may also suggest a mechanism for trans-synaptic tau propagation (92).

Phosphorylation clearly plays a role in the toxicity and localization of tau, however, its exact role in neurodegenerative disease is unknown and appears to be quite complicated. While hyperphosphorylated tau has been shown to have toxic results in the cell, increasing aggregation and abnormal tau localization (40, 90), dephosphorylated tau can also have harmful effects. Phosphorylated tau released into the medium of cultured neuroblastoma cells through muscarinic receptor activation that is dephosphorylated by tissue-non-specific alkaline phosphatase (TNAP) led to excitotoxicity, increasing calcium levels in nearby cells. Additionally, levels of TNAP are heightened in AD brains compared to control brains (93). Another study of primary cortical neurons also found that extracellular tau is largely dephosphorylated (94). Conversely, one study found that phosphorylation of tau increased its secretion from HeLa cells (95). Inflammation and activation of microglia has been shown to increase tau phosphorylation as well as aggregation, but is complicated by the fact that the opposite effect is seen in Aβ (96–99). The localization of tau in the cytosol, cell membrane, and the nucleus also appears to be important for tau toxicity, and is mediated by phosphorylation. Oxidative stress and heat shock induce the dephosphorylation of cytosolic tau and its transport into the nucleus. Once relocated to the nucleus, tau appears to protect neuronal DNA from damage under cell stress (100), which may be important in AD where DNA damage has been shown to occur (101). One possibility is that abnormal phosphorylation of tau in AD may prevent tau from being dephosphorylated and translocated to the nucleus to protect against DNA damage. The localization of tau to the cell membrane may also depend upon its dephosphorylation, as tau with lower levels of phosphorylation in its proline region was shown to be associated with the cell membrane, while phosphorylated tau was found in the cytoplasm (102, 103). However, interaction with membrane bound proteins, such as aforementioned fyn may stabilize association of phosphorylated tau with the membrane.

## Mechanism of Tau Propagation

The entry of prion proteins, Aβ, and other amyloid proteins into the cell via different mechanisms has been well-established (104–107). One hypothesis for amyloid oligomer toxicity and entrance into cells is through protein interaction with the cell membrane. One model suggests that oligomers embed themselves into the cell membrane and form pores. However, it appears as though the formation of pore-like annular protofibrils occurs through a separate pathway from fibril formation (108). An alternative model suggests that oligomers interact with lipid rafts in the bilayer, causing membrane thinning and increased membrane permeabilization, which may play a role in oligomer toxicity, allowing non-specific ion entrance, as well as leakage of cellular compartments. Several types of amyloid oligomers have been shown to increase membrane permeability, including Aβ, α-synuclein, and prion protein (109, 110, 107, 111). Tau has been reported to interact with the lipid rafts in the cell membrane and undergo conformational changes leading to membrane stress (112–115). Additionally, permeabilization of the membrane could mediate internalization of oligomers into the cell.

There has also been evidence for endocytosis as a route of amyloid entry into the cell. Propagation of α-synuclein, prion protein, Sup35, and Aβ has been shown to be associated with the endosomal pathway (116–118). One study found that tau aggregates colocalize with dextran in neural stem cells, implying that entry into the cell occurs via macropinocytosis. However, the aggregate type was not specifically tested (84). Aggregates identified specifically as tau oligomers colocalized with fluid-phase endocytosis marker, dextran, as well as with early endosomal marker, Rab5, and late endosomal/lysosomal marker, Lamp1. When endocytosis was inhibited with dynamin inhibitor, Dynasore, tau uptake was blocked, while inhibition of clathrin-mediated endocytosis with Pitstop2B did not impact internalization (83). These studies together suggest a mechanism for tau propagation in which tau is internalized via pinocytosis and enters the endosomal pathway. Tau can move through the endosomal pathway to the lysosome where toxic species may be degraded or recycled back to the cell membrane, where they may be released to be internalized by adjacent neurons. More research is needed to determine how membrane-enclosed tau oligomers are released inside of the cell, though it appears likely that the majority are degraded in the lysosome, while those that avoid degradation may cause the endosomal membrane to burst and be released in the cytoplasm, where they can seed aggregation of healthy tau (83). While clathrin-mediated endocytosis did not appear to be involved in tau propagation, endocytosis inhibitors are often found to be non-specific (119), and therefore, the possibility of other types of endocytosis in tau spread bears more study.

Receptor-mediated endocytosis could be another route of entry into the cell as amyloids have been reported to bind to cell surface receptors. Internalization of α-synuclein has been shown to be dependent upon receptor-mediated endocytosis, potentially through caveolin-mediated endocytosis (117). Additionally, Aβ binds to NMDA, α7 nicotinic acetylcholine, and APOE receptors, inducing receptor endocytosis (120–124). Aβ oligomers also bind cellular prion protein, PrP^c^, which is complexed with the Src tyrosine kinase, Fyn. This interaction has been shown to increase tau dysfunction and prevents native tau from binding to fyn (125). Under normal conditions, tau binds to fyn in oligodendrocytes (126) and in neurons, activating the Ras/MAPK pathway (103). Mutations to the microtubule binding region in tau lead to decreases in oligodendrocyte process number and length and disease-related missense mutations increase tau association with Fyn (127). Results indicate that the interaction between tau and fyn may be important for neurodegeneration, both through a loss in native tau interaction and through a gain in toxic tau function. Interaction with PrP^c^ complexed to fyn could also mediate tau entry into the cell as the PrP^c^ complex is associated with and endocytosed with caveolin (128). Tau may also enter the cell through a direct interaction with fyn. Lee et al. used a lamprey ABC tauopathy model in which tau is expressed in specifically identified ABC neurons to investigate the spread of tau. They report that tau phosphorylated at Y18, the site most commonly phosphorylated by fyn kinase, is associated with vesicular organelles. Additionally, when tau is overexpressed and localizes to the dendrite, dendritic vesicle accumulation is observed. Phosphorylated tau is colocalized with vesicles which bear resemblance to endosomes, as well as to fyn. Fyn has also been shown to colocalize with exosomes, suggesting a possible mechanism for fyn-tau transport in which fyn-associated tau is endocytosed, transported from early endosomes to late endosomal compartments, and then transported out of the cell via exosomes (91).

While one mechanism for tau oligomer release is through oligomer toxicity leading to cell death, causing the cell to lyse and release its contents (129), studies show that this likely does not account for the majority of tau release. In primary neurons treated with tau oligomers, extracellular tau only increases once levels as high as 40% cell death are reached, which does not correspond to physiological levels of cell death during the initial spread of neurodegenerative disease (130). Additionally, treatment with tau oligomers in primary neurons does not lead to significant levels of apoptosis (83). There has however been some evidence for non-apoptotic membrane blebbing as a possible secondary mechanism for tau release (91, 131).

Exocytosis has been implicated as a mechanism of amyloid spread as prion proteins and α-synuclein have been shown to be associated with exosomes in cell culture (132, 133). However, investigations of a similar mechanism for tau release have been unclear. Simón et al. found that when tau was overexpressed in kidney-derived cell lines, tau was secreted contained within membrane vesicles (129, 134). While tau secreted by neuroblastoma cells and tau in human CSF was found to be associated with exosomes in one study (135), another reported that tau was not detected in isolated exosomes from neuroblastoma cells (130). However, these studies used cell models where tau was overexpressed. In an attempt to approach more similar conditions to those seen physiologically, researchers cultured primary cortical neurons containing endogenous tau and found that tau was released by a mechanism unrelated to cell death and was regulated by AMPA receptor activation. Inhibition of synaptic vesicle release decreased extracellular tau, while tau was not found to be associated with exosomes, indicating that release of tau through traditional synaptic exocytosis following AMPA receptor activation may be one mechanism of tau release (94). Another study found that cells constitutively release tau which is not contained within a membrane under conditions inhibiting cell death (136). Therefore, more research is warranted to investigate the conditions under which tau is associated with exosomes and the specific tau conformations found in exosomes. Tunneling nanotubes – long, temporary channels that allow for long-distance transport between cells – have recently been discovered as a transport mechanism for prion protein (137). While they have not yet been studied directly in the context of tau, similarities between the spread of prions and tau suggest that tunneling nanotubes may be another potential mode of tau propagation meriting study.

## Conclusion

Determining how neurodegenerative tauopathies initiate and propagate toxic species will be crucial to finding a treatment for these diseases. Recent evidence suggests that tau oligomers, not NFTs, are the toxic tau species mediating the initiation, seeding, and propagation of neurodegenerative tauopathies and are the best target for anti-tau therapeutics. The mechanism by which tau seeding occurs remains to be elucidated, but oligomer-nucleated conformational induction, whereby native tau monomers are entirely converted to oligomers prior to aggregation into fibrils, appears to be a likely model. Tau oligomers can effectively enter cells, be transported intracellularly, and be released from cells to affect others. However, the mechanism by which propagation occurs is unclear. Tau likely enters the cell in one of two main ways, stressing the cell membrane or entering via endocytosis. Entrance through interaction with the membrane may occur through formation of pores or by interacting with lipid rafts causing membrane stress. Both macropinocytosis and receptor-mediated endocytosis have been implicated as possible mechanisms for tau entry. Tau secretion is likely not due simply to cell death, but may occur within exosomes, through synaptic vesicle release, or a non-traditional secretion pathway in which tau is not enclosed in a membrane. The elucidation of the mechanisms addressed will lead to a better understanding of neurodegenerative disease and may reveal new targets for treatment.

## Conflict of Interest Statement

The authors declare that the research was conducted in the absence of any commercial or financial relationships that could be construed as a potential conflict of interest.
